# Radiation-Associated Angiosarcoma After Breast Cancer: High Recurrence Rate and Poor Survival Despite Surgical Treatment with R0 Resection

**DOI:** 10.1245/s10434-012-2310-x

**Published:** 2012-03-31

**Authors:** Jojanneke M. Seinen, Emelie Styring, Vincent Verstappen, Fredrik Vult von Steyern, Anders Rydholm, Albert J. H. Suurmeijer, Harald J. Hoekstra

**Affiliations:** 1 Department of Surgical Oncology, University of Groningen University Medical Center, Groningen, The Netherlands; 2 Department of Pathology, University of Groningen University Medical Center, Groningen, The Netherlands; 3 Department of Orthopedics, Lund University, Lund University Hospital, Lund, Sweden

## Abstract

**Background:**

Secondary angiosarcoma of the breast is a rare but severe long-term complication of breast cancer treated with breast-conserving surgery and radiotherapy. We characterized a population-based cohort of patients with secondary angiosarcomas from two tertiary hospitals to investigate this complication with respect to surgical treatment and outcome.

**Methods:**

We identified 35 patients with a history of radiation for breast cancer that developed angiosarcoma in the irradiated field from 1990 to 2009. Of these, 31 underwent surgery and were included for analysis.

**Results:**

Angiosarcoma developed after median 7 years (range 3–25 years). R0 resection was obtained in 23 of 31 patients after primary treatment. Local recurrence developed in 19 patients after median 6 months (range 1–89 months). Regional and distant metastases occurred in 13 patients after median 17 months (range 2–50 months); nine which also had local recurrence. Patients whose local recurrence could be operated on had a better survival after treatment than those who were not considered for surgical treatment, median 34 months (range 6–84 months) compared with 6 months (range 5–24 months). The median disease-free survival and disease-specific survival was 16 and 37 months, respectively.

**Conclusions:**

Despite R0 resection, two-thirds of the patients developed a local recurrence. Survival among those with local recurrence was better if the patient could be treated with surgery. Overall, the prognosis was dismal and median DSS was just over 3 years.

Breast-conserving treatment (BCT) with radiotherapy has replaced mastectomy as the standard care for early-stage breast cancer in the last few decades.^1^ Radiotherapy is usually administered at a maximum of 50 Gy to the operated area, in some cases with an extra booster of 10–20 Gy to the tumor bed. The incidence of breast cancer is increasing; currently, it affects one in ten women in the western world. Accordingly, more secondary angiosarcomas have been reported, with a cumulative incidence of 0.9 per 1,000 breast cancer cases over 15 years.^2^ The development of secondary angiosarcoma has been linked to radiotherapy and lymphedema.^3^^–^ 5

Secondary angiosarcomas after BCT have an observed median latency period of ~4–8 years.^6^^–^ 10 Because of their rarity and seemingly harmless presentation, frequently comprising painless and bruise like skin lesions, both patients and doctors often neglect the initial symptoms and diagnosis is delayed. Patients often have localized, but multifocally growing, disease that is confined to the breast at diagnosis. Surgery is the mainstay of treatment and is usually performed with local resection or mastectomy. The risk of local recurrence and metastasis is high.^9^ There have been several studies of (neo-) adjuvant chemotherapy, but the effects remain unclear.^9^^,^ 11 ^,^ 12

The poor outcome of secondary angiosarcoma is well known, albeit mainly through numerous case reports.^8^^,^ 13 ^,^ 14 Few have looked at the surgical treatment and outcome.^15^ We have performed a 2-center retrospective study to characterize the disease with respect to treatment and outcome in a population-based cohort undergoing surgery with curative intent.

## Methods

### Cohort

We identified all female patients with histopathologically diagnosed angiosarcoma of the breast in the northern Dutch Health Care Region (1.8 million inhabitants) and the southern Swedish Health Care Region (1.7 million inhabitants). The northern Dutch part of the nationwide network and registry of histopathology and cytopathology in the Netherlands (PALGA) (since 1971) and the southern Swedish part of the national Swedish Cancer Registry (since 1958), and, in addition, the pathology registry (since 1960) were used to identify all cases within the populations. Patients with breast cancer diagnosed prior to angiosarcoma were selected (*n* = 50). Prerequisites used for this study were those proposed by Cahan et al.^16^ and modified by Arlen et al.^17^: (1) a sarcoma arising within the field of previous radiotherapy, (2) differing histology between the secondary sarcoma and primary tumor, and (3) at least a 3-year latency period between radiation therapy and development of the sarcoma. Under these criteria, 35 cases were identified from 1990 to 2009. Surgery with curative intent was performed on 31 patients. Data regarding the radiotherapy dose, type of surgery for the angiosarcoma, local recurrence, metastatic disease, and death were obtained from hospital and primary care records.

### Patient Characteristics

The median age of patients at the time of breast cancer diagnosis was 59 years (range 42–77 years). As part of breast cancer therapy, radiotherapy was administered at a median dose of 50 Gy (range 45–70 Gy). Nearly one-third of the patients received an extra radiation boost to the tumor bed, resulting in a total dose of 66–70 Gy.

### Outcome Measures

The endpoints of this study were disease-free survival (DFS), disease-specific survival (DSS), time to local recurrence, and time to distant metastasis. DFS was defined as the time from start of treatment to local recurrence, distant metastasis, or last follow-up. DSS was defined as time from diagnosis of secondary angiosarcoma to death due to angiosarcoma or last follow-up. Time to local recurrence was defined as the time from treatment to radiological or pathological confirmation of local recurrence. Time to distant metastasis was defined as the time from treatment of the secondary angiosarcoma to radiological or pathological confirmation of metastasis. In the text, surgical margins were defined as R1 (microscopically intralesional) or R0 (microscopically free margin).The data are presented as median and range, unless stated otherwise.

**Table 1:** Treatment and outcome for primary angiosarcoma disease

P	Primary operation	Reconstruction	Margins	(neo-) adjuvant treatment	Local recurrence	Metastasis
1	Mastectomy	STSG	R0	No	Yes	No
2	Mastectomy	STSG	R0^a^	No	Yes	No
3	Mastectomy	STSG	R0	No	No	Yes
4	Mastectomy	No	R0	No	No	No
5	Mastectomy	No	R0	No	No	No
6	Mastectomy	STSG	R0^a^	No	Yes	Yes
7	Mastectomy	No	R0	No	No	Yes
8	Mastectomy	STSG	R0	No	Yes	No
9	Mastectomy	No	R0	No	Yes	No
10	Local excision	No	R0^a^	No	Yes	No
11	Mastectomy	No	R0	No	No	No
12	Mastectomy	STSG	R0	No	Yes	Yes
13	Mastectomy	No	R0^a^	Radiotherapy	Yes	No
14	Local excision	STSG	R0	No	No	No
15	Mastectomy	Muscle flap	R2	Chemotherapy	Yes	Yes
16	Local excision	No	R0^a^	No	No	No
17	Mastectomy	STSG	R2	No	Yes	Yes
18	Mastectomy	No	R2	No	Yes	No
19	Local excision	STSG	R2	No	No	Yes
20	Local excision	No	R0	No	Yes	No
21	Mastectomy	STSG	R1	No	Yes	No
22	Mastectomy	No	R0	No	Yes	Yes
23	Mastectomy	No	R2	No	Yes	Yes
24	Mastectomy	No	R0	No	Yes	No
25	Mastectomy	STSG	R0	No	No	Yes
26	Mastectomy	STSG	R2	No	Yes	Yes
27	Mastectomy	STSG	R0	No	Yes	Yes
28	Mastectomy	No	R0	No	No	No
29	Mastectomy	Muscle flap + STSG	R0^a^	No	No	No
30	Local excision	No	R2	No	Yes	Yes
31	Local excision	Muscle flap + STSG	R0^a^	No	No	No

STSG split thickness skin graft

^a^After two surgeries for the primary tumor

### Statistical Analysis

The DFS and DSS were calculated according to the Kaplan–Meier method. All analyses were performed using SPSS software version 18 (released September 9, 2010; SPSS, Chicago, IL).

## Results

### Angiosarcoma Characteristics

**Fig. 1:**
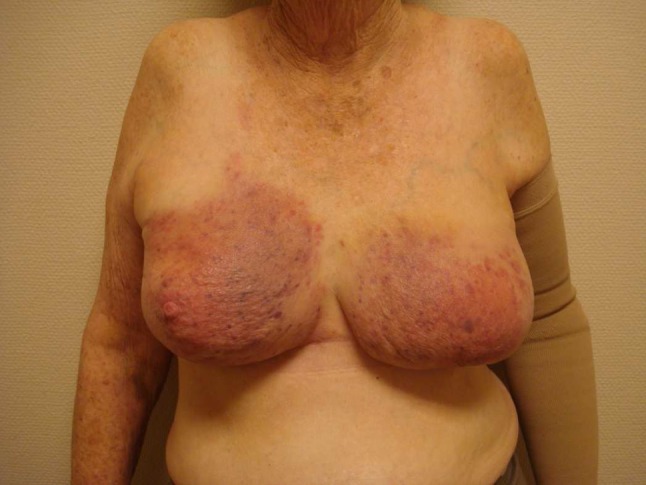
Patient with extensive angiosarcoma in both breasts

In 13 of 35 patients, angiosarcoma presented as a blue or red discoloration of the skin; in 14 patients, it started with a tumor, and in eight patients, both discoloration and tumor were present. The tumors had a median size of 4 cm (range 1–15 cm), with a multifocal appearance. The median time from the administration of radiotherapy to the development of angiosarcoma was 7 years (range 3–25 years), and the median age of the patients when the angiosarcoma was diagnosed was 67 years (range 47–89 years). Of the 35 patients, 31 underwent surgery with curative intent and were included in the survival analysis. The four patients who did not undergo surgery had metastatic or locally advanced disease (Fig. 1).

### Primary Treatment

The main surgical approaches used for resection of the primary tumor of the secondary angiosarcoma were local excision of the tumor (*n* = 7) (either due to previous mastectomy as part of breast cancer treatment or the local excision was performed outside our tertiary hospitals) and mastectomy (*n* = 24) (Table 1). In all cases operated on at the two tertiary centers, the aim was a minimum macroscopic margin of 2 cm. An R0 resection was achieved in 23 patients after primary treatment, seven patients were operated on twice for primary tumor. A total of 16 cases required reconstruction at treatment for the primary tumor, and the choice of reconstruction was tailored to the size of the defect. In 13 cases a split-thickness skin graft was used, in 1 case reconstruction with a pedicled flap of the latissimus dorsi and in 2 cases a combination of both techniques were used for reconstruction.

In addition to surgical treatment for the primary tumor, one patient received neoadjuvant chemotherapy (adriamycin 6 cycles) and one patient received adjuvant radiotherapy (50.4 Gy) after intralesional surgery.

In our series, mastectomy achieved R0 margins at higher rates than local resection (14 of 24 compared with 2 of 7). In addition, in three patients excision of all irradiated tissue was performed based on the radiotherapy field film, achieving R0 resection in 2 cases.

### Local Recurrence

After a median of 6 months (range 1–89 months), 19 patients developed a local recurrence. Of these, 14 had an initial R0 resection. In patients with local recurrence, 11 patients underwent surgery, which resulted in R0 resection in ten patients. Adjuvant radiotherapy was administered to one patient and adjuvant chemotherapy to one. Of the eight patients not undergoing surgery, one received radiotherapy and one both radiotherapy and chemotherapy (Table 2). The reason for not performing surgery in five of eight patients was concurrent metastasis, of whom four patients received chemotherapy. In the remaining three cases, extensive local disease was the reason not to perform surgery in 2 cases and in 1 case the reason was unknown. Patients who underwent surgery for local recurrence survived a median of 34 months (range 6–84 months) after surgery, compared with 6 months (5–24 months) for those who did not.

**Table 2:** Treatment and outcome for local recurrence and metastasis

N	Initial margin primary tumor	Time from treatment to LR (months)	Treatment for LR	Time from treatment to metastasis (months)	Treatment for metastasis	Survival after recurrence^a^ (months)
Local recurrence						
1	R0	5	Local excision	–	–	14
2	R0	2	Local excision	–	–	34
3	R0	89	Local excision	–	–	63
4	R0	40	Local excision	–	–	20
5	R0	18	Local excision	–	–	49
6	R0	5	Local excision	–	–	6
7	R0	4	Mastectomy	–	–	57
8	R0	22	Resection based on RT film; ifosfamid, doxorubicin (6 cycles)	–	–	84
9	R0	1	None	–	–	1
10	R1	86	None	–	–	2
Metastasis						
1	R0	–	–	17 (vertebrae)	RT 20 Gy	20
2	R0	–	–	2 (lung)	RT 41 Gy	16
3	R0	–	–	24 (axillary)	Resection	29
4	R0	–	–	50 (cerebral, abdominal)	None	3
Local recurrence + metastasis						
1	R0	6	–	23 (lung)	None	18
2	R0	8	Local excision	18 (vertebrae)	RT 8 Gy	16
3	R1	2	None	2 (contralateral breast)	Adriamycin (6 cycles); paclitaxel, bevacizumab (5 cycles); cyclofosfamid	24
4	R1	12	None	8 (axillary)	Resection, RT (dose unknown); adriamycin (5 cycles); fluorouracil, farmorubicin, cyclofosfamid (4 cycles)	14
5	R0	2	Local excision + RT 30 Gy	6 (thoracic wall into lungs)	Cisplatin	9
6	R1	2	–	2 (contralateral breast, sacrum)	None	1
7	R1	2	RT, dose unknown	2 (cerebral, abdominal)	Cisplatin	5
8	R0	18	None	17 (axillary, lung)	Resection + RT 45 Gy; doxorubicin + isofosfamid (1 cycle); gemzar, taxotere	8
9	R0	12	Local excision	41 (axillary)	Resection + RT 50 Gy; doxorubicin + ifosfamid	55

LR local recurrence, RT radiotherapy

^a^Recurrence: first appearing (local recurrence or distant metastasis)

### Metastatic Disease

There were nine patients who developed distant metastatic disease, and four patients developed regional metastatic disease after a median of 17 months (range 2–50 months). Of these patients nine also had a local recurrence. In the four patients with regional metastatic disease, resection of metastasis to lymph nodes was performed, which led to 1 case of R0 resection, 2 cases of R1 resection, and 1 case with unknown margins. Patients with regional metastasis who underwent surgery survived a median of 20 months (range 8–29 months) after surgery, compared with 5 months (range 1–24 months) for those with distant 

metastasis who did not undergo surgery. Of the 13 patients with metastases, six patients received chemotherapy and six patients underwent radiotherapy (Table 2).

### Survival

**Fig. 2:**
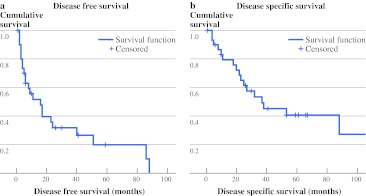
**a** DFS in months, **b** DSS in months. **a** There were patients alive without disease, these are among the censored cases

With a median follow-up of 27 months (range 1–151 months), 21 of 31 patients died; 17 of the deaths were due to angiosarcoma, three due to other diseases, and one patient died of unknown cause. There were ten patients still alive at last follow-up. Of these patients, nine had no evidence of disease after a median follow-up of 53 months (range 10–108 months). The remaining patient had local disease after 7 years. Of the patients with metastasis, one patient was still alive 2.5 years after surgery for regional metastasis. The three patients who underwent excision of all irradiated tissue, based on the radiation field films, with R0 margins were alive after 2, 2.5, and 9 years with no evidence of disease. For the total population, the median DFS and DSS was 16 and 37 months, respectively (Fig. 2a, b).

## Discussion

Although secondary angiosarcomas of the breast have an approximate incidence of only 0.9 per 1,000 breast cancer cases^2^, they are important because this disease has a very poor prognosis and is related to a previous medical treatment. In this study, two-thirds of the patients with secondary angiosarcoma presented with the typical blue/red discoloration of the skin, sometimes in combination with palpable tumor. The lesions can be single or multiple nodules, and papules or vesicles. One-third of the patients presented solely with a tumor; therefore, suspicion of secondary angiosarcoma should not rest only upon discoloration of the skin. In any patients with discoloration that does not disappear or tumor development within the radiation field, a needle or open biopsy should be performed. Mammography and/or MRI play only a limited role in diagnostics, but MRI may be slightly better at detecting an angiosarcoma.^18^^,^ 19 Since the first case of secondary angiosarcoma of the breast was described in 1987, the prognosis has remained poor, with a median survival of 1–3 years.^5^ In our population-based study, we found similar median survivals of 16 and 37 months for DFS and DSS, respectively. Only two of our 35 identified patients presented with metastatic angiosarcoma, both died of the disease within 6 months.

Compared with previously reported population-based studies (Fodor et al. 2006, *n* = 8; Hodgson et al. 2007, *n* = 31; and Marchal et al. 1999, *n* = 9)^6^^,^ 19 ^,^ 20, our cohort had similar median ages at the time of diagnosis of breast cancer (mean 60 years) and secondary angiosarcoma (mean 68 years). To the best of our knowledge, this is the equal largest study (*n* = 31) published for secondary angiosarcoma developing in the irradiated field, but the largest study focusing on surgical treatment and outcome.

Most secondary angiosarcomas present as localized disease without metastasis. Nevertheless, they are difficult to control and often recur locally.^9^ In our series, mastectomy achieved R0 margins at higher rates than local resection. In addition, three patients had mastectomy with resection of all irradiated tissue as primary treatment, R0 margins achieved in 2 cases and are alive after 2–9 years without evidence of disease.

Of the 31 patients who underwent surgery, the primary treatment resulted in R0 resections in 23 patients. Despite this, nearly two-thirds of these patients developed a local recurrence. We believe this to be due to the multifocal growth of angiosarcoma and tumor tissue left behind, even if the surgical margins are considered free. In this study, 11 of 19 patients with a local recurrence were considered eligible for surgery. The five patients with metastasis and three patients with locally extensive disease were not considered suitable for surgical treatment. The patients selected for surgery for local recurrence survived longer than those who did not. This difference we believe is due to different stages of tumor, and hence surgery has not necessarily made the difference. However, we conclude that local recurrence per se should not disqualify from aiming at surgery with curative intent.

In addition, four patients with lymph node metastases underwent surgery. Of these, two patients were without evidence of disease at the last follow-up, more than 2 years after surgery. The other two patients died of tumor after 8 and 14 months because of their angiosarcoma.

In other types of soft tissue sarcoma, adjuvant radiotherapy is frequently applied to improve local control. However, since secondary angiosarcomas develop within a field of radiation, usually the surrounding tissue has already received the maximum dose of radiation. In spite of this, one patient was treated with radiotherapy and initially responded well to therapy. After a few months, however, the disease progressed rapidly. A small single-center study (*n* = 14) that looked at hyperfractionated and accelerated radiotherapy (HART), with or without surgical resection, found a 64 % 5-year progression-free survival.^9^ Other studies reported a good response to (neo-) adjuvant treatment with paclitaxel in some patients.^12^^,^ 21 ^,^ 22 Interpretation of these results is hampered by the limited number of cases and the heterogeneity of the study populations. Therapies targeting angiogenesis through the vascular endothelial growth factor (VEGF) pathway and VEGF receptors (VEGFR) (e.g., sunitinib, sorafenib, and bevacizumab) have been promising in some patients with angiosarcoma; however, they clearly do not benefit a majority of patients.^23^

Another important field of study concerns the dose, extent, and duration of radiotherapy as part of breast-conserving therapy. Studies comparing hypofractionated and conventional fractionation of radiotherapy for breast cancer have been conducted, as have studies investigating radiotherapy for a quadrant of the breast instead of the entire breast.^24^^–^ 30 If changes in the dose, extent, and duration of radiotherapy are implemented, the risk of developing secondary angiosarcoma will have to be monitored closely.

In conclusion, the only chance of curative treatment for secondary angiosarcoma is extensive surgery, preferably with resection of all irradiated tissue performed. The rarity of the disease, its complex behavior, and the need for extensive surgery indicates that these tumors should be managed at a tertiary sarcoma center. Despite free margins, two-thirds of the patients in our study developed a local recurrence. Surgical intervention in a selected group improved survival for patients with local recurrence. However, the median DSS was still only 3 years.
